# Purification, Structural Characteristics, and Biological Activities of Exopolysaccharide Isolated From *Leuconostoc mesenteroides* SN-8

**DOI:** 10.3389/fmicb.2021.644226

**Published:** 2021-03-26

**Authors:** Junrui Wu, Danli Yan, Yumeng Liu, Xue Luo, Yang Li, Chengxu Cao, Mo Li, Qi Han, Cong Wang, Rina Wu, Lanwei Zhang

**Affiliations:** ^1^College of Food Science, Shenyang Agricultural University, Shenyang, China; ^2^College of Food Science and Engineering, Ocean University of China, Qingdao, China

**Keywords:** *Leuconostoc mesenteroides*, exopolysaccharide (EPS), characterization, antitumor activity, apoptosis, proliferation

## Abstract

In this study, a novel exopolysaccharide (EPS) was extracted from *Leuconostoc mesenteroides* Shen Nong’s (SN)-8 which can be obtained from Dajiang. After the purification step, EPS-8-2 was obtained with molecular weights of 1.46 × 10^5^ Da. The structural characterization of EPS indicated that the EPS belonged to the class polysaccharide, mainly composed of glucan and also contained certain mannose residues that were found to be connected by α-1,6 glycosidic bonds. Moreover, the results demonstrated that EPS displayed a significant capacity to scavenge free radical to some extent, and this anti-oxidant potential was found to be concentration dependent. The results further revealed that EPS displayed a significant inhibitory potential on the growth of HepG2 cells by promoting apoptosis and induced cell cycle arrest in G1 and G2 phases. Overall, these results suggested that EPS can be explored as a possible anti-cancer agent.

## Introduction

Exopolysaccharide (EPS) is a water-soluble polysaccharide that is generally secreted outside the cell wall during the growth and metabolism of few special microorganisms, and can be easily separated from the bacteria, and thereby secreted into the surrounding environment. EPS is a secondary metabolite derived from the microorganisms, and can be mainly divided into two categories: capsular polysaccharides that can attach to the cell wall of microorganisms to form capsules and mucus polysaccharides that can enter the culture medium to form mucus (Shukla et al., 2019). Many microorganisms, such as bacteria, algae, and fungi, have the ability to synthesize EPSs (Zhu et al., 2018). For instance, lactic acid bacteria (LAB) are generally considered as safe and edible that have been widely used in the food industry (Moradi et al., 2021). EPS is one of the active metabolites of LAB, which has been reported to exhibit potential therapeutic effects against cancer and immune related diseases (Rahbar Saadat et al., 2019; Xie et al., 2019a; Kostelac et al., 2021). Additionally, the wide-spread research in recent years, the possibility of industrial production of EPS has been explored and confirmed (Siddiqui et al., 2014), so the development potential of EPS for various applications may be substantial in the future. For example, in the field of food and cosmetics, few EPS with high thermal stability and solubility can be used effectively as a thickener or an emulsifier (Zhu et al., 2018; Rana and Upadhyay, 2020). According to previous reports, the various functional groups contained in EPS can contribute to the various pharmacological activities such as those related to antioxidant, antiviral, anti-inflammatory, regulation of the immune system, and anticancer in diverse models (Zhou et al., 2019).

Moreover, the EPS of *Phellinus badius* can also be lower blood sugar levels in rats with type 2 diabetes (Angelin and Kavitha, 2020; Riaz Rajoka et al., 2020; Sonawane et al., 2020). Therefore, EPS can be potentially used as a therapeutic agent to enhance immunity, and to treat diabetes as well as cancers such as colon cancer. Moreover, the EPS of *Bifidobacterium* can display significant resistance against allergies in OVA-gavaged mouse models by forming a physical barrier on the surface of small intestine and can be potentially used in the development of tissue scaffolds in the future (Luo et al., 2020). The development of microbial polysaccharides for possible use in the field of medicine needs further exploration and clinical verifications.

A variety of microorganisms can produce various EPSs, but the EPSs produced by different species of bacteria, fungi, and algae may display their own specific characteristics (Kumar et al., 2020). For instance, the EPS extracted from *Aerococcus uriaeequi* does not contain fructose as compared with the EPS of *Bacillus licheniformis* (Wang et al., 2018; Asgher et al., 2020). Although the characteristics of diverse EPSs obtained from the different bacterial genera are different, LAB have been extensively investigated in recent studies because of their high safety profile as well as associated economic benefits, and they are generally considered to be an important source of microorganisms. Moreover, in recent years, few novel purified EPSs of *Leuconostoc* spp. have been characterized by different spectroscopic techniques, for instance, *Leuconostoc pseudomesenteroides* (Yang et al., 2018), *Leuconostoc citreum* (Wang K. et al., 2020), and *Leuconostoc kimchii* (Yoon et al., 2000). *Leuconostoc* is one of the major species of LAB that can produce EPS. It can be derived from a wide range of natural sources, including human intestines, cheese, and other fermented products (Majumder et al., 2009). Additionally, *L. mesenteroides* strains have gained significant awareness for their application as potential probiotics (Tamime, 2002; Lee et al., 2016), which can effectively inhibit the inflammatory responses and survival of colon cancer cells by regulating the NF-κB/AKT/PTEN/MAPK pathways (Zununi Vahed et al., 2017). There are several prior studies elaborating the potential bioactivities of EPS extracted from *Leuconostoc* strains (Li L. et al., 2020), while there is little information available about the possible beneficial role of EPS isolated from *L. mesenteroides* to human health. Moreover, prior studies have also reported about the effects of EPS of *L. mesenteroides* on the immunomodulatory actions of the macrophages by effectively inhibiting the secretion of the pro-inflammatory cytokine IL-6 and increasing the secretion of the anti-inflammatory cytokine IL-10 and their possible applications in treatment (Kook et al., 2019). Overall, as their potential utilization continues to increase rapidly, exploring novel laboratory sources and characterizing their EPS still remain an urgent and meaningful task.

In order to enrich the number of EPS-producing strains, to perform structural and biochemical characterization, and to strengthen the findings related to the biological activities of EPS as well as to explore their potential applications in food, cosmetics, pharmaceuticals, and other fields, this study was performed in different steps using various procedures: First, EPS produced by *L. mesenteroides* Shen Nong’s (SN)-8 was systematically isolated and identified from Dajiang. Preliminary laboratory tests have confirmed that the quality of Dajiang was related to the metabolites of microbial community (Wu et al., 2018; Xie et al., 2019b; An et al., 2021). The capsule polysaccharides produced by *Bacillus velezensis* SN-1, which were isolated and screened from the Da-jiang in our laboratory, have the ability of anti-oxidation and anti-tumor (Cao et al., 2020). Thereafter, the molecular mass, monosaccharide composition, and elemental composition of *L. mesenteroides* SN-8 EPS were analyzed. The structural properties were determined using Fourier transform infrared (FT-IR) and nuclear magnetic resonance (NMR) spectra. Moreover, the anti-tumoral and antioxidant properties of *L. mesenteroides* SN-8 EPS were also investigated *in vitro* using hepatocellular carcinoma cell line, HepG2. The findings clearly suggested that *L. mesenteroides* SN-8 EPS may display beneficial effects that can be potentially used in the food industry and improving medical health.

## Materials and Methods

### Culture Preparation

*Leuconostoc mesenteroides* SN-8 was isolated from Dajiang (FuXin, China) by natural fermentation process, and its sequence was compared to that in the databases of the National Center for Biotechnology Information (NCBI; GenBank accession number(s): SUB9040225 Leuconostoc MW629943). Mannose, abinose, fucose, fructose, glucose, xylose, and galactose were procured from MREDA (China). All other reagents used are analytically pure. α-Diphenyl-β-picrylhydrazyl (DPPH) and 3-(4,5-dimethylthiazol-2-yl)-2,5-diphenyltetrazolium bromide (MTT) was bought from Macklin Co., Ltd. (Shengyang, China). Sephadex G-100 cellulose was purchased from Dulai Bio-Technology Co., Ltd. (Nanjing, China). Dimethyl sulfoxide (DMSO) and phosphate buffered saline (PBS) were obtained from Sale EI Chemical Reagent Co., Ltd. (Shengyang, China).

### Extraction and Purification of EPS

The strain was cultured in a Shaker Incubator (80 rpm) at 30°C for 48 h in MRS medium containing 45 g/L sucrose, and the inoculation quantity was 2% (v/v) of the seed medium culture. Thereafter, EPS was separated and purified by slightly modifying the procedure of Purama et al. (2009). First, the strain cells were carefully removed by centrifugation at 4,000 *g* for 40 min at 4°C. Then, threefold volume of 95% (v/v) cold ethanol was added to the cell-free culture to precipitate EPS and the mixture was left at 4°C for overnight incubation. After centrifugation at 10,000 *g* at 4°C for 60 min, crude EPS was recovered, suspended in pure water, and stirred with a magnetic stirrer until it dissolved completely. Thereafter, 10% (v/v) trichloroacetic acid (TCA) was added to the EPS solution to make the final concentration at 5%, and the mixture was stored at 4°C overnight. The proteins were removed by centrifugation at 4°C, 11,200 *g* for 60 min, then three volumes of 95% (v/v) cold ethanol were mixed with the supernatant, and the mixture was left overnight at 4°C. The EPS was centrifuged at 10,000 *g* for 60 min at 4°C and then dissolved in ultrapure water. Thereafter EPS solution was subjected to dialysis with dialysis bag (MW cut-off at 14,000) to eliminate the small molecules or ions at 4°C for 2 days. The EPS solution was further purified with a Sephadex G-100 chromatography column (1.6 cm × 50 cm). The elution buffer used was deionized water, with a flow rate maintained at 0.2 mL/min. The collections were performed using an ultraviolet (UV) spectrophotometer (Shanghai, China), and the wavelength was monitored at 220 nm. Finally, the purified EPS was concentrated and lyophilized before using it for the various experiments.

### Ultraviolet Spectroscopy Analysis

The polysaccharide solution with a concentration of 1 mg/mL was prepared by adding distilled water to 5 mg quantity of EPS. An optimal amount of EPS solution was placed in the colorimetric dish, and UV spectroscopic analysis was performed between 180 and 350 nm. The appearance of UV absorption peak at 260 and 280 nm was observed to determine the extent of purity of EPS.

### Molecular Weight and Monosaccharide Composition Analysis

The monosaccharide composition of EPS was further analyzed by gas chromatography (GC). In short, the purified EPS (10 mg) was hydrolyzed with 2 mL of 2 mol/L trifluoroacetic acid (TFA) at 120°C for 6 h to remove residual TFA in the hydrolyzate by evaporation process. Thereafter based on the methods of Yang et al. (2015), the dry hydrolyzed products were converted into acetylated derivatives. Then, GC analysis was performed using an HP-5 capillary column (30 m × 0.32 mm; I.d 0.25 m) (Agilent Technologies Co., Ltd., United States) on an instrument equipped with a flame ionization detector (FID). The operating conditions used were as follows: the injection temperature was 250°C; the injection volume was 3 μL; the detector temperature was maintained at 250°C; and the division ratio 3:1. The monosaccharide composition of EPS was determined by comparing with the standard sugars (mannose, arabinose, fucose, fructose, glucose, xylose, and galactose). The molecular weight was identified according to the method previously reported by Purama et al. (2009).

### FT-IR Spectroscopy Analysis

The functional groups of purified SN-8 EPS were further analyzed using FT-IR spectrometer (Tensor 27, Bruker, Germany). For this, 1 mg of dry EPS powder was mixed with 100 mg of potassium bromide (KBr) and continuously pressed to obtain a metal mold. Thereafter, in the frequency range of 400–4,000 cm^–1^, the FT-IR spectra of the particles were characterized with a resolution of 1 cm (Xing et al., 2018).

### NMR Analysis of EPS

The structural properties of *L. mesenteroides* SN-8 were determined through the 1D ^1^H and ^13^C NMR spectra and 2D heteronuclear single quantum coherence (HSQC) spectra. The *L. mesenteroides* SN-8 was detected by NMR spectroscopy (Avance III, Bruker, United States). The purification of EPS (30–50 mg) was thereafter exchanged three times with deuterium trioxide, then dissolved in 0.55 mL deuterium oxide (D_2_O), and placed in the NMR tubes. Mestrenova software was applied for data analysis.

### Thermodynamic Stability Analysis

The differential scanning calorimetry (DSC) and thermal analysis (TGA) was measured under atmospheric pressured by a DTA-DSC thermal analyzer (STA44gf3, Netzsch, Germany). The lyophilized EPS samples were placed into aluminum oxide (Al_2_O_3_) crucible. The experiments were carried out in the temperature range of 25–800°C, and the TG-DSC thermogram was obtained based on the relationship between the weight loss rate and heat flow and temperature.

### *In vitro* Antioxidant Activity of EPS

#### Scavenging Activity of DPPH Radical

The free radical scavenging activity of DPPH is an accepted method for screening the antioxidant activities. DPPH (2,2-diphenyl-1-picryl-hydrazyl-hydrate) method is an antioxidant assay that is primarily based on electron-transfer procedure that can generate a violet solution in ethanol. It was used here after slightly modifying the previously described procedure by Wang et al. (2019). In other words, 65 μmol/L DPPH radical was dissolved in ethanol that was used as the analytical reagent. The reaction was initiated by mixing 1 mL of NAOSs of different concentrations with DPPH reagent (5 mL). Then, the mixture was swirled and incubated in a dark environment at room temperature for 30 min. A UV spectrophotometer was used to bleach DPPH radicals at 517 nm. About 1 mL of the NAOS solution in ethanol was used as colorimetric reagent. The control group (*A*_0_) was a mixture of 1 mL water and 5 mL DPPH. All the samples were analyzed in triplicate, and the results were averaged. DPPH free radical scavenging activity (%) was calculated using the following formula:

(1) $$\mathrm{DPPH}\mathrm{free}\mathrm{radical}\mathrm{scavenging}\mathrm{activity}\left(\%\right)=\frac{A_{0}-A_{s\,a\,m\,p\,l\,e}}{A_{0}}\times 100$$

#### Scavenging Activity of ABTS Radical

2,2′-Azinobis-(3-ethylbenzothiazoline-6-sulfonic acid) radical scavenging activity was measured based on Matsuzaki’s method (Matsuzaki et al., 2017). The maximum absorption wavelength of ABTS radical ions is 734 nm, and hence the absorbance at this particular wavelength is used to detect the concentration of ABTS radical ions. If the absorbance decreases, it primarily indicates that ABTS has been effectively removed from the base ion. First, ABTS solution was obtained as described: 7 mM ABTS was mixed with 2.4 mM potassium persulfate (K_2_S_2_O_8_) cultured in dark for 12–16 h and then the ABTS solution was diluted to 0.700 ± 0.020 (OD_734nm_). Then, NAOS samples were dissolved in deionized water and the different concentrations were obtained for the experiment (15–35 mg/mL). After that, the NAOS solution (1 mL) was added to the ABTS solution (3 mL) and incubated at 37°C for 10 min. Thereafter, the absorbance was measured at 735 nm. The ABTS radical scavenging activity (%) was calculated using the following formula:

(2) $$\mathrm{ABTS}\mathrm{free}\mathrm{radical}\mathrm{scavenging}\mathrm{activity}\left(\%\right)=\frac{A_{0}-A_{s\,a\,m\,p\,l\,e}}{A_{0}}\times 100$$

Where *A*_0_ was the absorption of the control group, Asample was the absorption of sample group.

#### Scavenging Activity of ⋅OH

⋅OH scavenging activity was measured according to the previously described method (Feng et al., 2018). Salicylic acid can collect the hydroxyl radical in the Fenton reaction system and potentially form a purple 2,3-dihydroxybenzoic acid, and hence measuring its absorbance can indicate the exact amount of hydroxyl radicals. For this assay, 100 μL of ferrous sulfate (FeSO_4_) solution (9 mM) was mixed with 100 μL of salicylic acid–ethanol (9 mM), and 700 μL of polysaccharide solution (0.5–8.0 mg/mL). Thereafter 100 μL hydrogen peroxide (9 mM) was added to the solution mixture and incubated at 37°C for 30 min. The absorbance was recorded at 510 nm. The hydroxyl radical scavenging activity was calculated based on the following formula:

(3) $$\mathrm{Scavenging}\mathrm{activity}\left(\%\right)=\frac{A_{0}-\left(A_{1}-A\,2\right)}{A_{0}}\times 100$$

Where *A*_0_ was the absorbance of the blank, when the distilled water was used instead of the sample solution, *A*_1_ was the absorbance of the sample solution, and *A*_2_ was the absorbance of the control, when the distilled water was used instead of the H_2_O_2_ solution.

### *In vitro* Antitumor Activities of EPS

The half maximum inhibitory concentration (IC50) of EPS against human liver cancer cells (HepG2) was determined by MTT assay to evaluate the possible cytotoxic activity of EPS (Majumder et al., 2009; Capek et al., 2011). The succinate dehydrogenase in the mitochondria of living cells can effectively reduce the exogenous MTT to water-insoluble blue-purple crystal formazan and deposit it in the living cells, whereas the dead cells cannot display this property. Briefly, HepG2 cells were maintained in minimum essential medium (MEM), containing 10% fetal calf serum, 1% (v/v) glutamine, and 50 μg/mL of gentamycin sulfate [37°C, 5% (v/v) CO_2_ atmosphere]. The cultured logarithmic growing cells were seeded in 96-well plates at a density of 1 × 10^5^ cells/mL (100 μL/well). Thereafter the cells were incubated for 24 h, followed by the treatment with different concentrations of EPS (1,000, 2,000, and 5,000 μg/mL) or CdCl_2_ (60 μM) for 24 h, and then MTT reagent (5 mg/mL, 10 μL) was added 4 h before the end of the reaction. Finally, 100 μL of DMSO solution was used to replace the supernatant. The negative control groups consisted of cells that were cultured in the absence of EPS and CdCl_2_.

The absorbance was measured at 570 nm, and the percentage of cell proliferation was calculated based on the following formula:

(4) $$\text{Viability}\left(\%\right)=\left[1-\frac{A_{t\,r\,e\,a\,t\,e\,d}-A_{b\,l\,a\,n\,k}}{A_{c\,o\,n\,t\,r\,o\,l}-A_{b\,l\,a\,n\,k}}\right]\times 100$$

Where *A*_*control*_ and *A*_*blank*_ were the absorbance of the system without addition of EPS and cells.

In order to analyze the possible effects of EPS on the colony-forming ability, the cells were seeded into six-well plates at a density of 1 × 10^5^ cells/cell and EPS (1,000, 2,000, and 5,000 μg/mL). After incubation for 24 h, the medium was aspirated and an equal amount of fresh medium was added, including EPS of different concentrations. The culture was thereafter continued for further 48 h, and then observed upside down under the TS 100 microscope (Japan).

### Cell Cycle Analysis

The cell cycle analysis was performed according to a previously published method by Yu et al. (2019), Briefly, HepG2 cells (4 × 10^5^/well) were inoculated into a six-well plate with different concentrations of EPS (200, 600, 2,000, and 5,000 μg/mL) to study cell cycle progression. The cells were then harvested 24 h later, fixed overnight with 70% cold ethanol, and stored at –20°C. Thereafter the fixed cells were washed with PBS and this step was repeated three times. After that, the cells were incubated in 50 μg/mL RNase at 37°C for 30 min to degrade the RNA present in the sample. Finally, the cells were stained with propidium iodide (PI) dye (50 μg/mL) which can bind to DNA, at 4°C for 10 min in dark. The samples were then detected by flow cytometer (FACS Callibur, Becton Dickinson, United States) and analyzed with the ModFit LT software.

### Statistical Analysis

The data were represented as mean ± standard errors of three independent experiments. The statistical analysis was performed using SPSS 19.0 software. The differences were considered significant when *p*-value was <0.05.

## Results and Discussion

### Strain Identification and Purification of EPS

**FIGURE 1:**
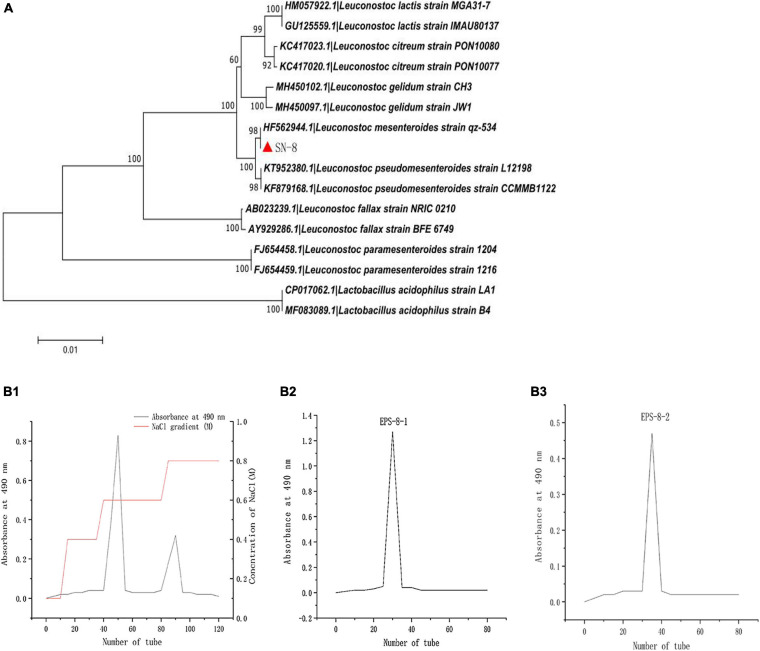
Phylogenetic tree of *L. mesenteroides* SN-8, based on 16S rDNA sequences **(A)** and Sebhadex G-100 chromatogram of *L. mesenteroiaes* SN-8 EPS **(B1–B3)**.

The strain named as SN-8 was identified from *L. mesenteroides* by physio-chemical, morphological, and 16S rDNA sequence analysis (Figure 1A). After chromatographic purification with Sephadex G-100 dextran gel, a pure polysaccharide dried sample was obtained for the experiments. It can be clearly seen from Figure 1B1 that two distinct peaks appeared in different time periods, thereby indicating that the polysaccharide may contain two different components, which can be divided into EPS-8-1 and EPS-8-2 (Figures 1B2,B3). EPS-8-2 was observed to possess better stability and hence may be more conducive for large-scale production and processing in the future; thus, it was chosen for subsequent tests.

### Purity Analysis of EPS

The EPS produced by *L. mesenteroiaes* SN-8 was scanned using UV spectrometer in the wavelength range of 180–350 nm. The scanning result has been shown in Supplementary Figure 1. The purified EPS-8-2 had no obvious UV absorption at 260 and 280 nm, thereby indicating that the sample obtained had a high purity.

### Determination of EPS Molecular Mass and Analysis of Monosaccharide Composition

**FIGURE 2:**
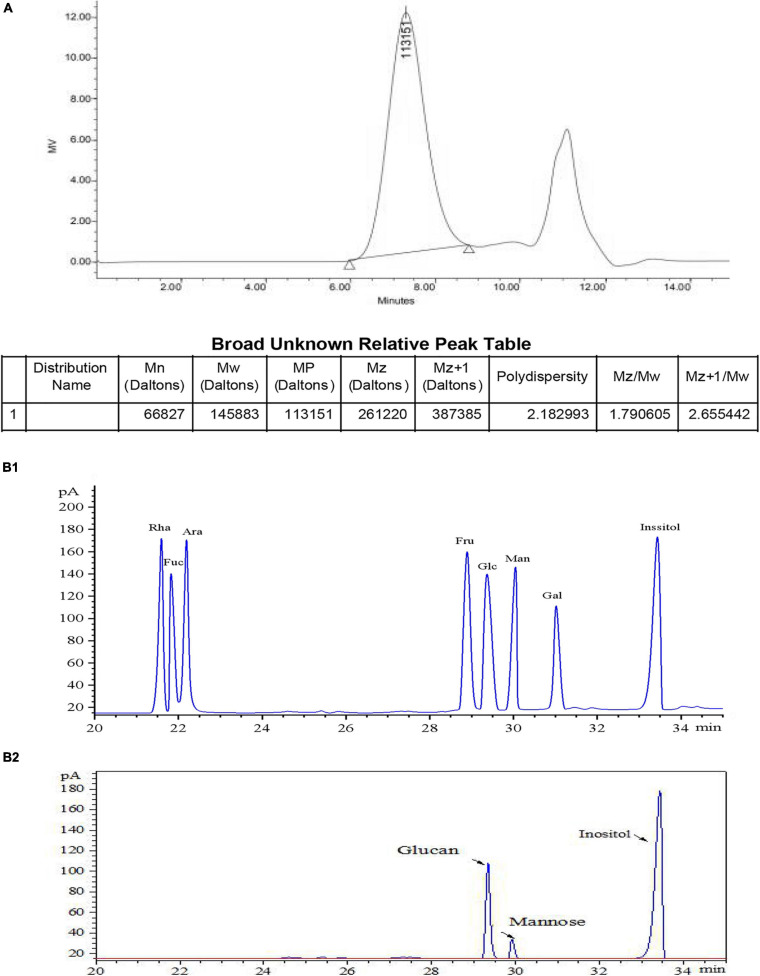
Determination of molecular mass of EPS-S-2 **(A)**, standard monosaccharide by GC **(B1)**, and monosaccharide composition of EPS-8-2 by GC **(B2)**.

The molecular weight of EPS was thereafter determined by High perfomance size exclusion chromatography (HPSEC) analysis. Two symmetrical and narrow peaks were observed in the HPSEC profile (Figure 2A), and this result validated the high purity of EPS. A linear regression curve was generated using dextran standards, and the estimated molecular weights of EPS were found to be 1.46 × 10^5^ Da, which was higher than that of *Cynanchum auriculatum Royle ex Wight* (0.83 × 10^6^ Da) (Yu et al., 2019) and lower than that of *L. pseudomesenteroides* YF32 (5.54 × 10^6^ Da) (Sardari et al., 2017) and *Weissella cibaria* YB-1 (3.89 × 10^6^ Da) (Zhang et al., 2019). Subsequently, the monosaccharide composition of EPS analyzed by GC and compared with the mixed standard solution (Figure 2B1) was studied (Figure 2B2). The results of GC analysis further indicated that the purified EPS was primarily composed of dextran and contained few mannitose residues upon comparison with the retention time of different standard monosaccharides.

### EPS Structural Analysis

Every molecule possesses a specific frequency that enables them to rotate or vibrate at this particular frequency, which is consistent with the reported working mode of FT-IR (Lin et al., 2009). The infrared spectrum result of SN-8 EPS

has been shown in Supplementary Figure 2. The sharp and broad peak around 3,406.59 cm^–1^ could be attributed to O–H stretching vibration. The signal at 1,652.69 cm^–1^ indicated that C=O stretching vibration was possibly the characteristic absorption of polysaccharides (Kaur and Kapoor, 2008; Majumder et al., 2009). The absorption peaks at 1400.58 cm^–1^ were found to be caused by C-H stretching vibration (Yu et al., 2019). The signal peak in 1053.17 cm^–1^ clearly demonstrated that the EPS contained α-(1→6) glucosidic bond (Gan et al., 2011). These results clearly showed that EPS possessed the basic skeleton and functional groups of a typical polysaccharide structure.

### Thermodynamic Research

The study of thermodynamic properties can aid one to better understand whether a certain substance may possess a wider potential commercial application value, thereby leading to the rapid development of new products (Gan et al., 2011). The thermal decomposition process of different substances may also vary, but usually can include the following steps: dehydration, decomposition, and reorganization of chemical bonds. Supplementary Figure 3 clearly shows the thermodynamic analysis results of *L. mesenteroiaes* SN-8 EPS. It can be clearly observed from the figure that as the temperature increases, the quality of the EPS-8-2 can be affected significantly (Jadaun et al., 2019). The range was found to be limited (about 4.5%), while the mass of EPS-8-2 began to decrease at around 100°C. The substantial loss of water may also cause the quality to change at this stage, at around 300°C, due to the significant increase in temperature. The EPS appeared to have undergone thermal decomposition and its quality had also decreased significantly (about 68.7%); and it was observed that upon increasing the temperature, the decomposition occurred to a greater extent (Wang Y. et al., 2020). Since the main decomposition process has occurred at about 300°C, there was less significant residual material at this stage, and so the mass loss at this stage was also expected to be less (about 5.8%). The above experimental results clearly showed that the EPS produced by *L. mesenteroiaes* SN-8 was observed to be significantly resistant to high temperatures and thus possibly suitable for use in the food processing industry.

### NMR Spectroscopy Analysis

**FIGURE 3:**
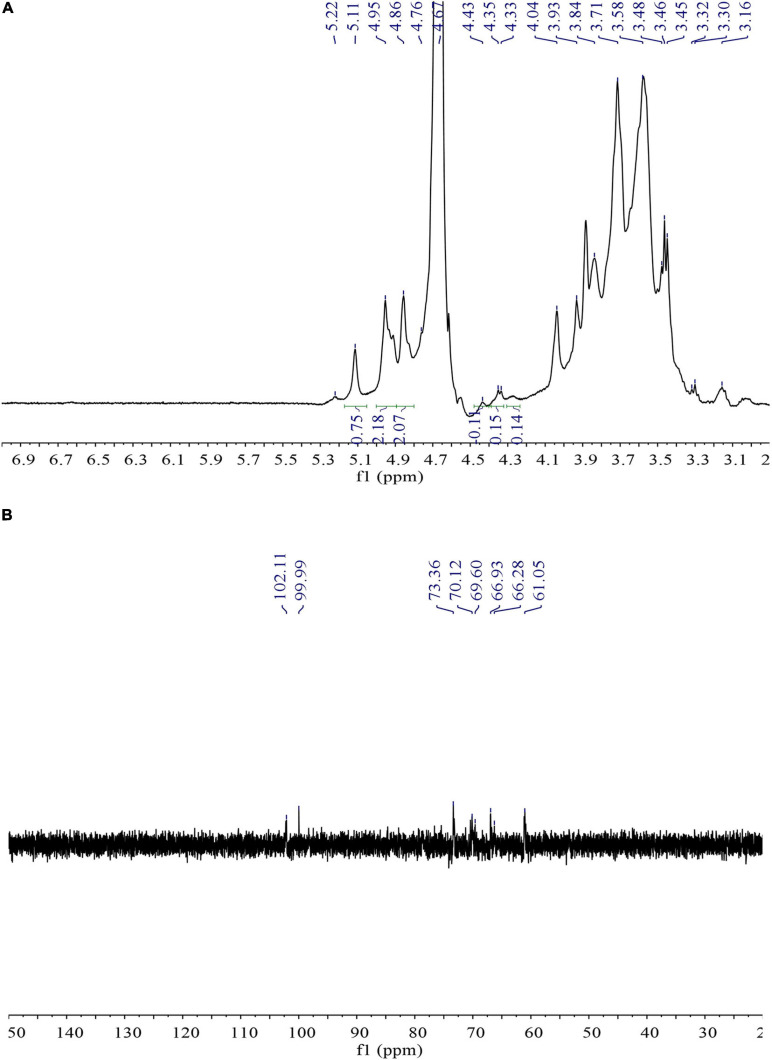
1D ^1^H **(A)** and ^13^C **(B)** NMR spectra of *L. mesenteroiaes* SN-8 EPS.

Nuclear magnetic resonance spectroscopy can serve as an effective tool for the analysis of complex material structures. There have been few reports related to the possible use of NMR to detect the structure of bacterial EPS (Gan et al., 2011). In this study, ^1^H NMR spectrum was elegantly used to analyze the glycosidic bond configuration of *L. mesenteroiaes* SN-8 EPS sample. It can be clearly seen from Figure 3A that only a high intensity proton signal peak appeared distinctly at 4.67 ppm in the anomeric proton region, which indicated that the sample primarily contains α-(1→6)-linked D-pyranose residue (Jadaun et al., 2019). This observation was found to be relatively consistent with the characteristic absorption peak of the sample at 1,053.17 cm^–1^ in the FT-IR spectrum.

^13^C NMR spectroscopy can be mainly used to determine the type and configuration of glycosidic bonds in the polysaccharides (Xu et al., 2019). Figure 3B is the ^13^C NMR spectrum of *L. mesenteroiaes* SN-8 EPS sample. It can be clearly observed from the figure that a high-intensity signal appeared distinctly at the anomeric carbon region at 99.99 ppm, thereby representing an α-(1→6) glycoside. The high signal corresponded to the anomeric proton signal at 4.67 ppm in the ^1^H NMR spectrum. In addition, the signal peaks that appeared at different chemical shifts 73.36, 70.12, and 69.60 ppm, respectively, represented C-3, C-5, and C-4 glucose residues (Mende et al., 2016). The signal peak at 66.28 ppm could represent the C-6 signal of the glucose unit, thus indicating that the two glucose units in the EPS main chain were actually connected by α-(1→6) glycosidic bonds. The clear absence of other signal peaks in the 75–85 ppm region indicated that EPS did not contain additional branched chains and is primarily composed of an α configuration pyranose.

### Results of *in vitro* Antioxidant Studies

#### Scavenging Effect of DPPH

**FIGURE 4:**
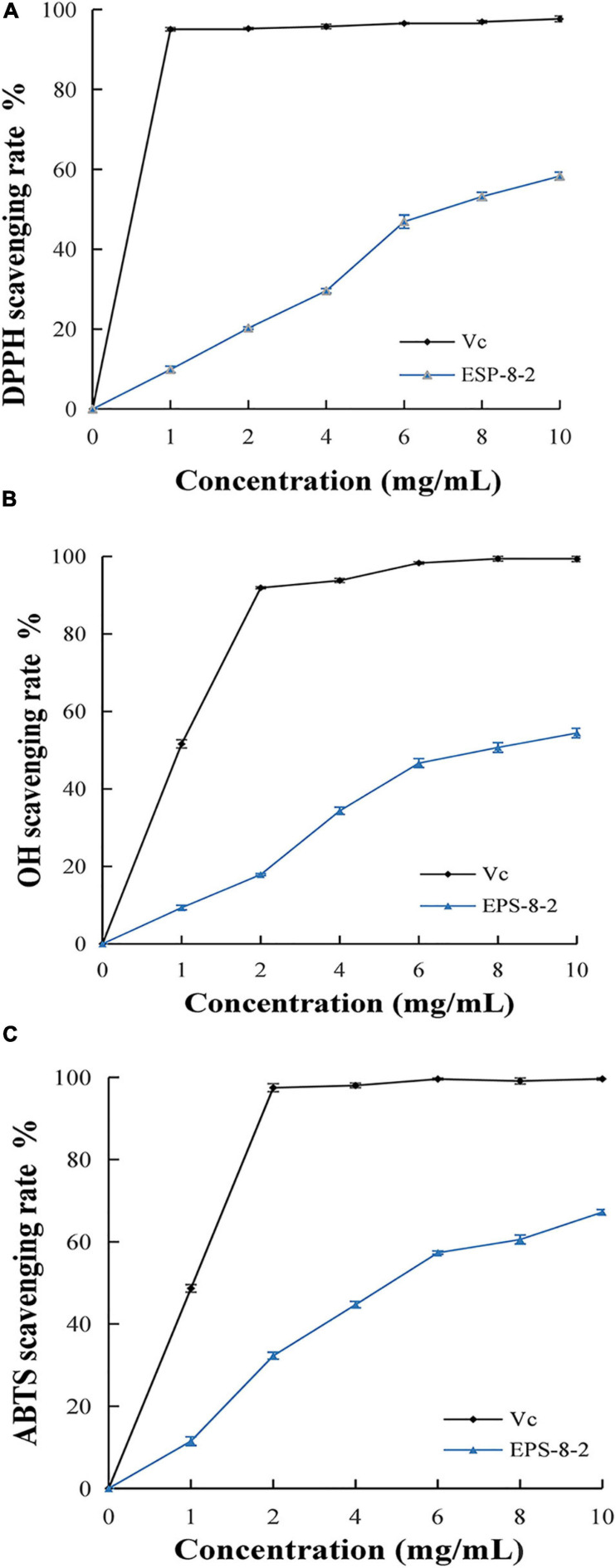
*In vitro* antioxidant activity of EPS from *L. mesenteroides* SN-8 using ascorbic acid (Vc) as reference, respectively. **(A)** DPPH radical scavenging ability; **(B)** hydroxyl radical scavenging ability; **(C)** ABTS radical scavenging activity.

The analysis of DPPH free radical scavenging rate is one of the most widely used assays for determining the scavenging activity of the various natural compounds (Wang Y. et al., 2020). As shown in Figure 4A, EPS-8-2 exhibited significant radical scavenging ability that reached up to 57.42 ± 1.38% at the concentrations range varying from 0 to 10 mg/mL. However, at all concentrations used in this study, its scavenging activity was significantly weaker than that of Vitamin C (Vc).

#### Scavenging Effects of ⋅OH

As a result of its fluid structure, the cell membrane can easily lose charge and the hydroxyl radicals can freely enter the cell membranes and cause tissue damage (Zhou et al., 2013). Thus, removal of the special free radicals can prevent tissue injury (Zhang et al., 2019). The scavenging activities of EPS-8-2 on hydroxyl radicals have been shown in Figure 4B. It exhibited a significant scavenging effect on hydroxyl radicals. At 10 mg/mL, the scavenging ability of EPS-8-2 reached to 72.4 ± 0.32%, comparable to Vc (95.6 ± 0.06%). This result is in agreement with the previously published research results of polysaccharide from *Artocarpus heterophyllus* Lam. pulp that too displayed a powerful hydroxyl radicals scavenging activity, but markedly feebler than that of VC (Mende et al., 2016). In summary, EPS-8-2 could possibly function as effective antioxidants and thus may limit cellular damage caused by free radicals.

#### Scavenging Effect of ABTS+

There is a significant evidence regarding the involvement of reactive oxygen species in the pathophysiology of several chronic diseases (Valko et al., 2007). Hence, the reducing ability of novel drug candidates can serve as an important index about their potential antioxidant activities and pharmacological efficacies against different diseases (Zhang et al., 2019). As shown in Figure 4C, the reduction ability of the sample increased significantly with a proportional increase in polysaccharide and V_*C*_ concentration. The higher absorbance value observed thereby indicated relatively a more effective reduction ability of the samples. Interestingly, the molecular weight of polysaccharides also affected the reducing power, for instance, the lower was the molecular weight, the more stronger reducing power was displayed by the polysaccharides (Kassim et al., 2010). Therefore, compared with the reducing power of Vc at 10.0 mg/mL, the reducing power of EPS-8-2 was noted to be significantly lesser. Similarly, the polysaccharides isolated from *Artocarpus heterophyllus*, pumpkin, and *Arctium lappa* displayed a very low reducing capacity (Mende et al., 2016).

### Antitumor Activity of EPS *in vitro*

**FIGURE 5:**
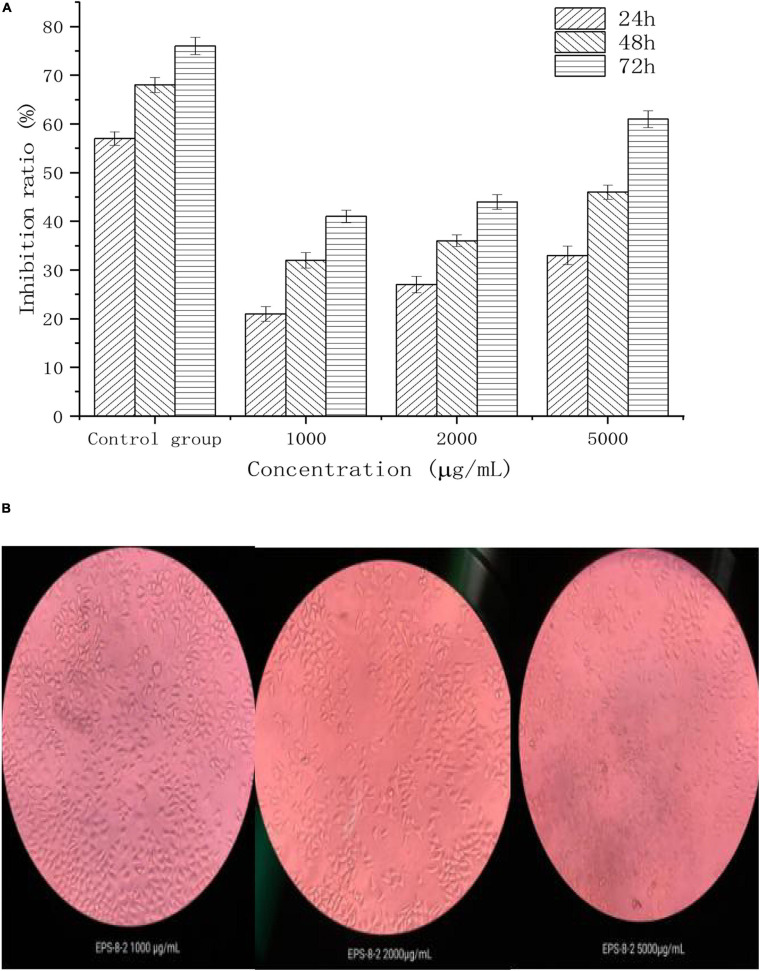
Antitumor effects of EPS-8-2 **(A)** on HepG-2 cells. All values were expressed as means ± standard deviation (SD) of three replications **(B)**.

We next evaluated the anticancer activities of EPS under *in vitro* settings. Dajiang is a common fermented food found in the northern China. *L. mesenteroides* SN-8 is a common LAB isolated from Dajiang. A large number of studies have shown that the EPS produced by LAB can exhibit diverse pharmacological activities such as acting as anticancer, antibacterial, and antioxidant, and thus it may have significant therapeutic potential. Moreover, EPS produced by LAB alone may not display significant toxicity toward the normal cells (Sirin and Aslim, 2020). Figure 5 shows the inhibitory effect of different concentrations (1,000, 2,000, and 5,000 μg/mL) of EPS-8-2 against the growth of HepG2 cells. It was observed that with the increasing EPS concentration, the growth inhibition rate of HepG2 cells showed a clear upward trend. For instance, at a concentration of 5,000 μg/mL, the inhibition rate reached the maximal value (*p* < 0.05). In addition, we also observed the possible effect of EPS-8-2 on the density and morphology of HepG2 cells using an inverted microscope. We noted that the morphology of HepG2 cells was relatively single, complete morphology and tightly connected in control group. After incubation with EPS-8-2 for 48 h, it was observed that the number of HepG2 cells gradually decreased, and the intercellular space became more distinct. Meanwhile, a large number of cells expanded and contracted, and cell debris increased (Pan and Mei, 2010; Miao et al., 2014). The findings indicated that EPS-8-2 can play an inhibitory role in the formation and aggregation of HepG2 cells, which was also consistent with the observations of MTT experiment results. The biological activity of EPS has been reported to be related to the activity of its functional groups (such as hydroxyl, amino, carbonyl, and carboxyl groups) (Sathiyanarayanan et al., 2017). EPS-8-2 has been found to have hydroxyl and carbonyl groups by FT-IR, so it can display significant anticancer activities. The molecular weight of polysaccharides has also been linked to their potential anticancer effects. A number of studies have shown that the anticancer effect of polysaccharides with high molecular weight is significantly stronger than that of polysaccharides with a molecular weight of 5 × 10^3^–1 × 10^4^ Da (Zekovic et al., 2005; Zhang et al., 2017). The molecular weight of the purified EPS-8-2 was 1.46 × 10^5^ Da, and thus it was able to demonstrate significant anticancer activities.

### Effect of EPS on the Cell Cycle Progression

In order to investigate whether cell cycle termination was the major cause of inhibition of growth potential HepG2 cells induced by EPS, PI staining method was used to detect the distribution of PI stained HepG2 cells in different phases of the cell cycle. PI can penetrate the damaged cell membranes and bind to DNA to produce red fluorescence, which can be analyzed by flow cytometry (Niu et al., 2015). The G1 and G2 phases represent the early and late stages of mitotic DNA replication, respectively (Israels and Israels, 2001). The results in Table 1 clearly showed that the ratio of the cells in G1 and G2 phases was significantly decreased at a concentration of more than 200 μg/mL EPS-8-2 as compared with the untreated group. The ratio of G1 and G2 phase in control group was 51.24 ± 0.74 and 8.97 ± 0.16%, and that of 200 μg/mL EPS-8-2 was 32.94 ± 0.52 and 6.54 ± 0.14%. These observations suggested that EPS-8-2 could induce substantial G1 and G2 phase arrest in HepG2 cells. The decreased percentages of HepG2 cells in the G1 phase clearly revealed that EPS-8-2 could induce apoptosis of cancer cells effectively. It has been previously reported that a polysaccharide isolated from *Polygonatum cyrtonema* Hua could effectively block the G2/M phase of cell cycle by regulating the expression of *CDK1* and *CyclinB1* genes in Hela cells (Li Y. et al., 2020).

**TABLE 1:** The effect of EPS on the proportion of G1 and G2 in HepG2 cell cycle.

	The proportion of G1 phase (%)	The proportion of G2 phase (%)
Control	51.24 ± 0.74	8.97 ± 0.16
200 μg/mL	32.94 ± 0.52	6.54 ± 0.14
600 μg/mL	30.61 ± 0.51	5.83 ± 0.08
2,000 μg/mL	27.58 ± 0.42	5.27 ± 0.12
5,000 μg/mL	24.87 ± 0.49	4.86 ± 0.10

*Values are mean ± SD of three different observations (*n* = 3).*

## Conclusion

In the early stage of the experiment, a strain with high EPS production was isolated and screened from the naturally fermented soybean paste using viscosity measurement and polysaccharide content determination. After morphological observation, physiological and biochemical tests, and 16SrDNA sequence analysis, it was identified as *L. mesenteroides* and named *L. mesenteroides* SN-8. In this study, the EPS from *L. mesenteroiae*s SN-8 was isolated and characterized. EPS was purified with a molecular weight of 1.46 × 10^5^ Da. The results of GC, FTIR, and NMR clearly indicated that the EPS belonged to the class polysaccharide with a highly branched main chain of dextran with (1→6) connections, and contained few mannose residues as well. Moreover, the findings revealed that EPS exhibited significant antioxidant activity (DPPH, hydroxyl, and ABTS radical) in a concentration-dependent manner in various *in vitro* assays. Furthermore, EPS showed significant antitumor potential by exerting marked inhibitory effects on the growth of HepG2 tumor cells and could also induce G1 phase arrest in HepG2 cells. Overall, these results suggested that EPS may possess a potent antitumor activity *in vitro* and can be further explored as a potential anti-cancer agent in preclinical settings. The relationship between EPS structure and function will be further studied in the future.

## Data Availability Statement

The original contributions presented in the study are included in the article/Supplementary Material, further inquiries can be directed to the corresponding author/s.

## Author Contributions

JW contributed to the experimental design, performed the statistical analysis, and wrote the manuscript. DY performed the statistical analysis and wrote the manuscript. YL, CC, ML, QH, and CW contributed to visualization, software, and validation. XL, RW, and LZ contributed to manuscript revision. All authors contributed to the article and approved the submitted version.

## Conflict of Interest

The authors declare that the research was conducted in the absence of any commercial or financial relationships that could be construed as a potential conflict of interest.

## Supplementary Material

Supplementary Figure 1 UV–vis spectroscopy scanning of puried EPS-8-2.

Supplementary Figure 2 Infrared scanning results of exopolysaccharide (EPS). Different absorption peaks indicate that the EPS probably has a different structure.

Supplementary Figure 3 Differential scanning calorimetry thermogram of EPS-S-2. The experiment was processed at a temperature range of 0–700°C.
