# Supplementary figures and images for: Purification, Structural Characteristics, and Biological Activities of Exopolysaccharide Isolated From Leuconostoc mesenteroides SN-8
Source: Front Microbiol. 2021 Mar 26;12:644226. doi: 10.3389/fmicb.2021.644226 (PMC8033024; doi:10.3389/fmicb.2021.644226)

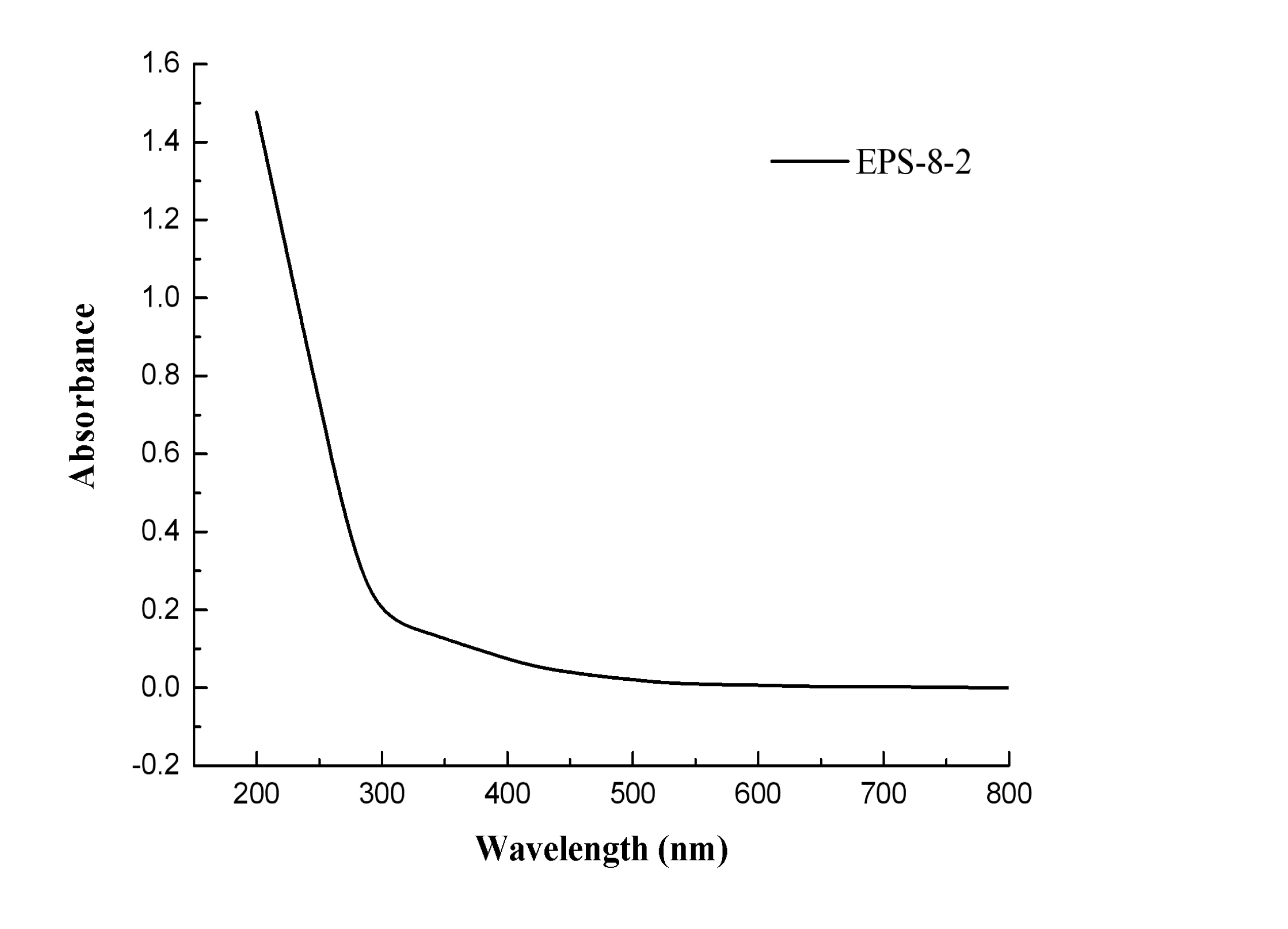

Supplement: Supplementary Figure 1 — UV–vis spectroscopy scanning of puried EPS-8-2. [file Image_1.JPEG]

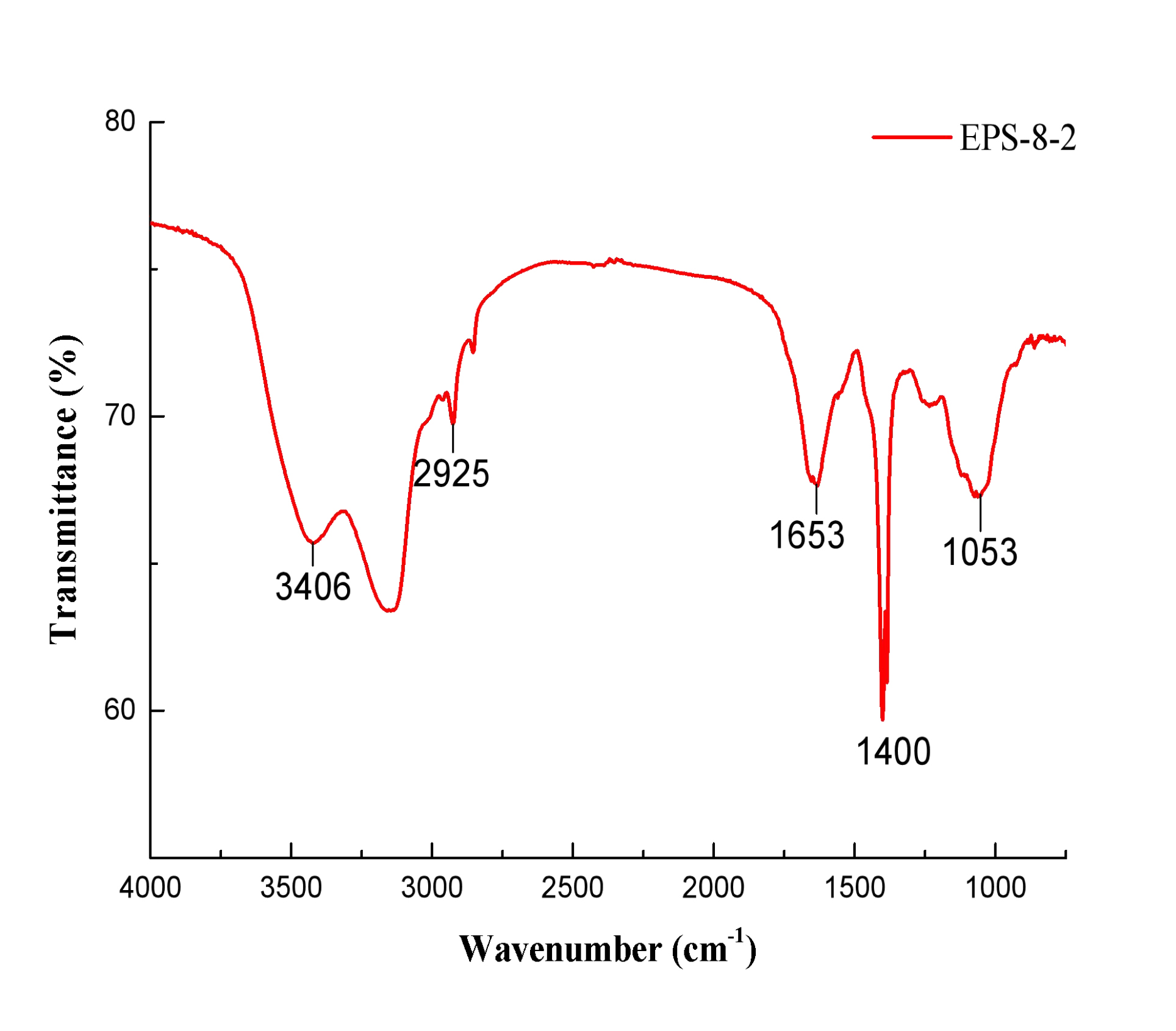

Supplement: Supplementary Figure 2 — Infrared scanning results of exopolysaccharide (EPS). Different absorption peaks indicate that the EPS probably has a different structure. [file Image_2.JPEG]

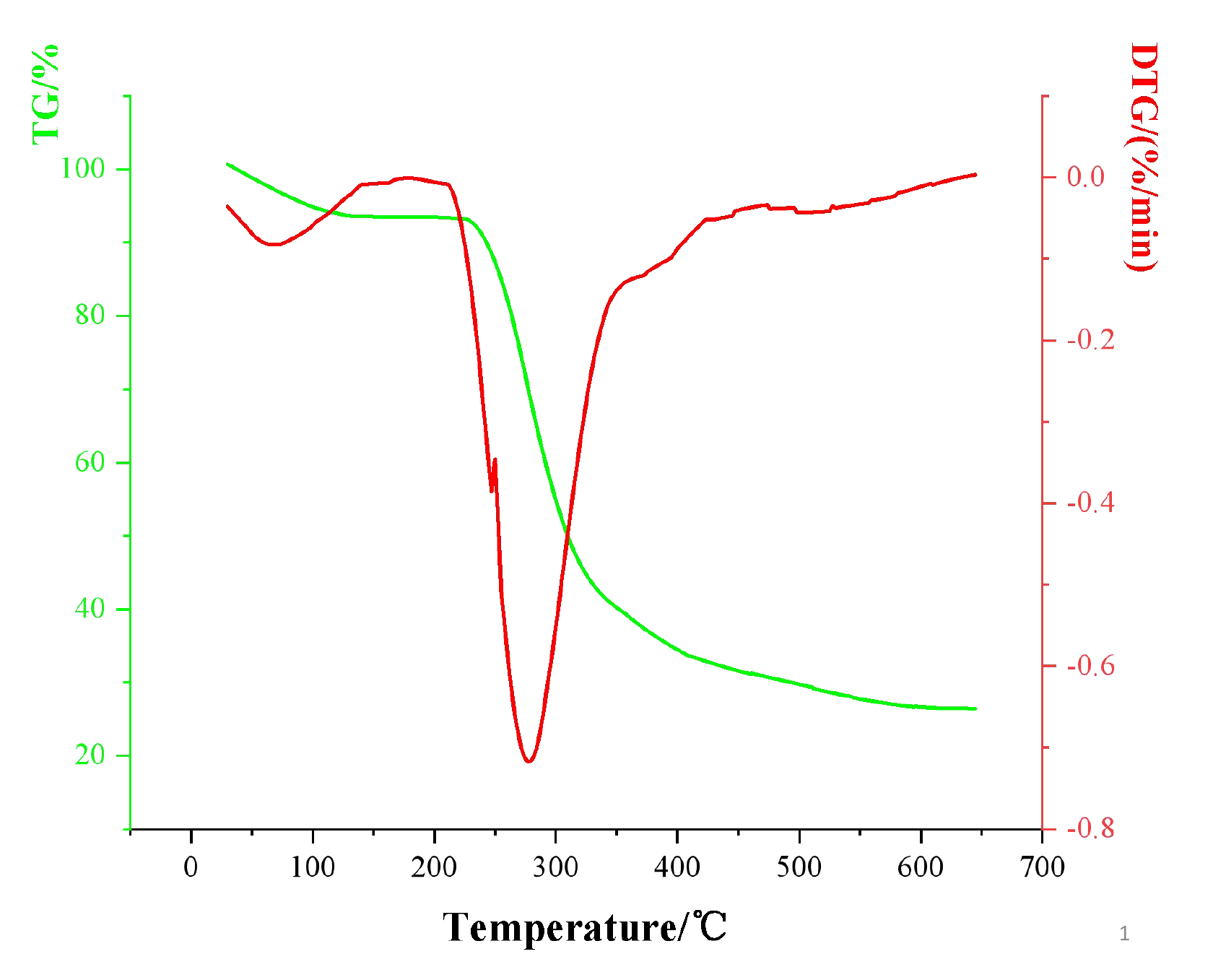

Supplement: Supplementary Figure 3 — Differential scanning calorimetry thermogram of EPS-S-2. The experiment was processed at a temperature range of 0–700°C. [file Image_3.JPEG]
